# Hemoglobin Levels and Platelet Counts after Hysteroscopy Due to Abnormal Uterine Bleeding

**DOI:** 10.3390/diagnostics12030594

**Published:** 2022-02-25

**Authors:** Katarzyna Jobda, Łukasz Szeszko, Grzegorz Wróbel, Marta Głuchowska, Joanna Krupińska, Artur Szeszko, Beata Makaruk, Przemysław Oszukowski, Paweł Zieliński

**Affiliations:** 1 Department of Gynecology and Obstetrics, Międzyleski Specialist Hospital, 04-749 Warsaw, Poland; kjobda@wp.pl; 2 Obstetrics and Gynecology Ward Provincial Specialist Hospital, 21-500 Biała Podlaska, Poland; lukasz.szeszko@op.pl; 3 Department of Anatomy Collegium Medicum, Jan Kochanowski University, 25-317 Kielce, Poland; pawel.zielinski@ujk.edu.pl; 4 Collegium Medicum, Jan Kochanowski University, 25-317 Kielce, Poland; gluchowskam95@gmail.com (M.G.); joannaa.krupinska@gmail.com (J.K.); arturszeszko@gmail.com (A.S.); 5 The Józef Pilsudski University of Physical Education, 00-809 Warsaw, Poland; beata.makaruk@awf.edu.pl; 6 Department of Obstetrics, Gynecology and Oncological Gynecology, Clinic of Obstetrics and Perinatology, Medical University of Łódz, 93-316 Łódź, Poland; przemyslaw.oszukowski@umed.lodz.pl

**Keywords:** abnormal uterine bleeding, hysteroscopy, hemoglobin, gynecology, obstetrics, endometrial polyp, adenomyosis, leiomyomas

## Abstract

Abnormal uterine bleeding (AUB) is a condition defined as all uterine bleeding that differs from physiological menstruation. The etiology of AUB has been classified by the International Federation of Gynecology and Obstetrics (FIGO). It includes structural categories, such as endometrial polyps, adenomyosis, leiomyomas, hyperplasia, and malignant neoplasms, and non-structural categories, i.e., hemorrhages due to congenital and acquired coagulopathies, ovarian dysfunction, disorders of the local endometrial hemostasis mechanism with normal organ structure, iatrogenic causes, and due to other poorly defined causes. This is a retrospective study based on the medical data of a group of 543 women aged 21–88 years (52.81 ± 11.79) (*p* < 0.01) hospitalized at the Gynecology and Obstetrics Department in Biała Podlaska, Poland. These patients underwent an hysteroscopy procedure due to excessive uterine bleeding of varied, FIGO-divided etiology. The results show the dependence of postoperative hemoglobin and platelet count on the etiology of bleeding and the age of the women. The majority of patients had normal hemoglobin and platelet counts after the procedure, while moderate anemia was the most common disorder. It occurred most frequently in patients undergoing hysteroscopy due to heavy menstrual bleeding.

## 1. Introduction

Abnormal uterine bleeding (AUB) is the collective term for all uterine bleeding that differs from physiological menstruation in regularity, frequency, duration or volume [1]. It is a common condition found in gynecology and can affect patients’ quality of life physically, emotionally, professionally, and sexually. About 30% of women will experience this problem in their lifetime [2,3]. The type of treatment, which needs to be applied depends on the cause, duration, and severity of the bleeding, and in some cases, surgical intervention is required [4]. In order to systematize the causes and qualification for the appropriate treatment of AUB, the International Federation of Gynecology and Obstetrics (FIGO) has developed a classification under the acronym PALM COEIN, which includes structural and nonstructural etiologies of abnormal uterine bleeding [5,6,7,8].

As category AUB-*p*, benign hyperplasia of endometrial glands which generally protrude into the uterine cavity, such as endometrial polyps, are described. It is a relatively common condition that may regress spontaneously, but in symptomatic cases, can be effectively and safely treated by hysteroscopic excision [9]. Hysteroscopy is a minimally invasive procedure that allows for the visualization of the entire uterine cavity. New small-diameter hysteroscopes are equipped with a surgical channel, which makes it possible to treat benign diseases and to perform biopsies in a relatively short time without anesthesia and under ambulatory conditions. It is now considered the gold standard in diagnostics and treatment of intrauterine pathologies [10].

AUB-A includes adenomyosis, a condition in which abnormally located endometrial glands and stroma are present within the myometrium. Based on ultrasound data, the prevalence of adenomyosis is estimated to be 20.9% in the general population, while data from histological examination hysteroscopy materials indicate values ranging from 10–35%. The disease requires a management plan, such as control of bleeding, pain, fertility, and pregnancy success. To date, no international guidelines have been established for the qualification for medical or surgical treatment of adenomyosis [11]. The AUB-L category concerns uterine fibroids (myomas, leiomyomas), which are the most common benign uterine tumors in premenopausal women [1]. It is estimated that over their lifetime, uterine fibroids will occur in 70% of women. There is a wide range of treatment options for uterine fibroids, many of which are still researched, but currently the most effective method is hysterectomy [12]. Endometrial hyperplasia and malignancies are category AUB-M and should always be considered in the differential diagnosis of abnormal uterine bleeding [1]. Patients with early diagnosed disease have a good prognosis, but for those with recurrent endometrial cancer or metastases, the median overall survival remains short [13]. AUB-C describes hemorrhages due to congenital or acquired coagulopathy. Hereditary coagulopathies, of which von Willebrand disease is the most common, occur in 5–25% of women with heavy menstrual bleeding. The most common causes of acquired coagulopathies are leukemia, aplastic anemia, hepatic or renal failure, sepsis, disseminated intravascular coagulation syndrome, and medications that affect coagulation [14]. Ovarian dysfunction is identified as AUB-O. It is typically observed during menarche and perimenopause, but can occur at any time during the reproductive life. It is diagnosed by excluding other organic or functional causes [15]. Primary disorders of the local endometrial hemostasis mechanism, which coexists with a structurally normal uterus and regular menstruation are categorized as AUB-E [1]. AUB-I category includes iatrogenic causes and AUB-N consist of other, unclassified or poorly defined disease entities and rare uterine abnormalities [6,16].

The possibility of hysteroscopic visualization of the uterine cavity has revolutionized the diagnosis and treatment of AUB. It is a safe, well-tolerated, simple, and reliable procedure that has broad functions in the management of excessive uterine bleeding [17].

Massive blood loss can lead to anemia, coagulopathy, acidosis, and hypovolemic shock [18]. The initial aim of treatment is to stabilize the patient’s hemodynamics. In a stable patient, the purpose is to control the bleeding, identify the cause, and limit future episodes [19].

The purpose of this study was to evaluate hematologic parameters, such as hemoglobin and platelet level after an hysteroscopy procedure in patients with abnormal uterine bleeding.

## 2. Materials and Methods

### 2.1. Patients and Reasons for Hospitalization

This is a retrospective study based on data collected during the year 2019. The medical records of 543 women, aged 21–88 years (52.81 ± 11.8 years) (*p* < 0.01) hospitalized in the Gynecology and Obstetrics Department of the Provincial Specialist Hospital in Biała Podlaska, Poland, were used for the study. All of the patients underwent hysteroscopy due to AUB. During the examination, a blood sample was taken from each patient and hemoglobin and platelet levels were indicated. Due to the lack of a suitable group of patients who could constitute a control group, no groups were formed. Patients included in the study were divided into groups based on the reason for hysteroscopy:

Group 1: Pathological bleeding from the uterus—212 patients (39.04%)
Group 2: Endometrial hyperplasia—147 patients (27.07%)
Group 3: Endometrial polyp—104 patients (19.15%)
Group 4: Heavy menstrual bleeding—62 patients (11.42%)
Group 5: Myomatosis—18 patients (3.3%)

Women were also grouped according to their pre- or postmenopausal status:

Group A: Premenopausal age—240 patients (44.2%)
Group B: Postmenopausal age (>50 years)—303 patients (55.8%)

Blood samples were taken from each patient after the hysteroscopy procedure, from which the level of hemoglobin and platelets was determined. The laboratory examination was performed in the diagnostic laboratory of the Provincial Specialist Hospital in Biała Podlaska, Poland. Hemoglobin reference values have been defined as the range of 12–16 g/dL. Moderate anemia was diagnosed at hemoglobin concentrations between 12–10 g/dL and moderate severe anemia for values between 10–8 g/dL. For platelets, the reference values were within the range of 150–400 G/L.

### 2.2. Statistical Analyses

Statistical analysis was performed using Statistica 13.3 software (TIBCO Software Inc., Palo Alto, CA, USA). In order to test the normality of the distribution of quantitative variables, the Shapiro–Wilk test was used. The non-parametric Kruskal–Wallis test and Spearman’s rho correlation were used to investigate the necessary dependencies. All of the tests were performed at the significance level of 0.05. The Guilford classification was used to assess the strength of the correlation.

## 3. Results

The analysis carried out among all of the 543 patients showed that the average hemoglobin concentration was 13.27 ± 5.46 g/dL (H = 21.00; *p* < 0.001), while the average platelet count was 244.81 ± 65.46 Giga/L (H = 15.56; *p* < 0.01). In 17.25% (94 patients), the hemoglobin level was below the reference range, while the platelet count was lower in 4.04% (22 patients). In the group of all premenopausal patients (240 women), the mean hemoglobin concentration was 12.82 ± 1.36 g/dL (*p* < 0.001). In addition, 18.33% (44 patients) of this group had anemia. The mean value of the number of blood platelets was 253.82 ± 63.49 Giga/L (*p* < 0.01), while 2.5% (6 patients) were below the reference values. Three hundred and four postmenopausal women were enrolled in the study. Among this group, the mean hemoglobin value was 13.56 ± 1.11 g/dL (*p* < 0.01). Moreover, 16.83% (51 women) were below the reference values. Platelet counts had a mean value of 239.4 ± 60.13 Giga/L (*p* < 0.01) and 5.28% (16 women) had thrombocytopenia. The upper limit of the reference range for hemoglobin was exceeded by two patients (0.37%), but these were very slight deviations (0.1 and 0.3 g/dL above the upper limit of normal). An elevated platelet count occurred in 1.47% (8 patients).

**Figure 1:**
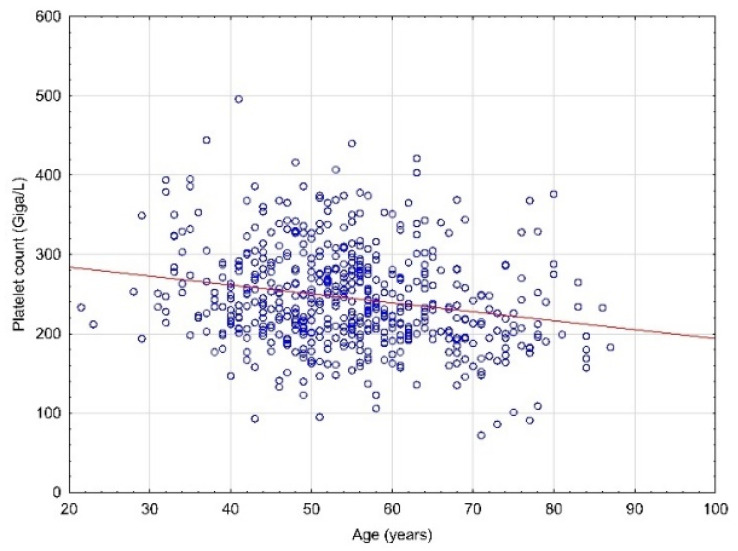
Correlation between the age of the patients and the values of the platelet count.

In the case of hemoglobin concentration value, a slight positive correlation was found (r = 0.21; *p* < 0.0001), and in the case of platelet count, a weak negative correlation was found (r = −0.22; *p* < 0.0001—Figure 1).

### 3.1. Pathological Uterine Bleeding

**Figure 2:**
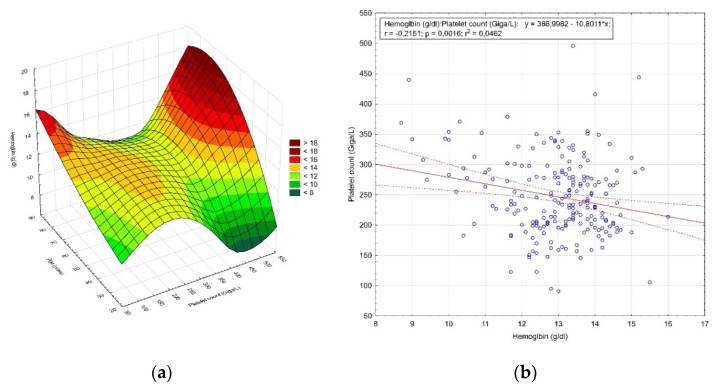
The results of the analysis of patients qualified for hysterectomy due to excessive uterine bleeding. (**a**) Distribution of the hemoglobin concentration value and the number of platelets by age; (**b**) correlation between the hemoglobin concentration value and the number of platelets.

Two hundred and twelve patients were qualified for hysterectomy due to excessive uterine bleeding. The age of the patients ranged from 27 to 85 years (52.7 ± 10.1 years) (*p* = 0.017). In addition, 40.1% (85 patients) were of premenopausal age and 59.9% (127 patients) of postmenopausal age. Among these patients, the mean hemoglobin level was 12.96 ± 1.28 g/dL (*p* < 0.0001) and 17.92% (38 patients) were below the lower limit of the reference range. Moreover, 16.5% (35 patients) had moderate anemia, while 1.41% (3 patients) had moderately severe anemia. The mean platelet count for this group was 246.98 ± 64.28 Giga/L (*p* = 0.002). Eight patients (3.77%) were below the reference range. The distribution of the hemoglobin concentration value and the number of platelets by age is shown in Figure 2a, while the correlation between the hemoglobin concentration value and the number of platelets is shown in Figure 2b.

### 3.2. Endometrial Hyperplasia

**Figure 3:**
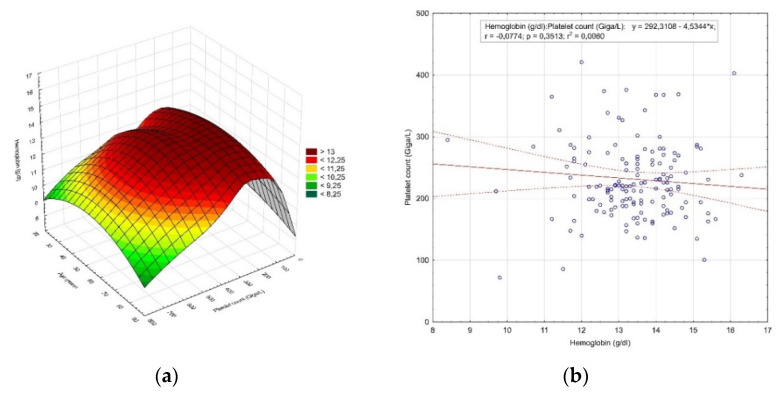
The results of the analysis of patients hospitalized for endometrial hyperplasia. (**a**) Distribution of the hemoglobin concentration value and the number of platelets by age; (**b**) correlation between the hemoglobin concentration value and the number of platelets.

One hundred and forty seven women aged 34–88 (60.5 ± 10.9 years) (*p* = 0.167) were hospitalized for endometrial hyperplasia. In this group, 18.4% (27 women) were premenopausal, while 81.6% (120 women) were postmenopausal. The mean hemoglobin value in this group was 13.38 ± 1.22 g/dL (*p* = 0.002). In addition, 12.24% (18 patients) were below the normal range, of which 11.56% (17 patients) had moderate anemia and 0.68% (1 patient) had moderately severe anemia. Platelet count averaged 231.61 ± 71.52 Giga/L (*p* < 0.0001) and 6.12% (9 women) were below the reference range. The distribution of the hemoglobin concentration value and the number of platelets by age is shown in Figure 3a, while the correlation between the hemoglobin concentration value and the number of platelets is shown in Figure 3b.

### 3.3. Endometrial Polyps

**Figure 4:**
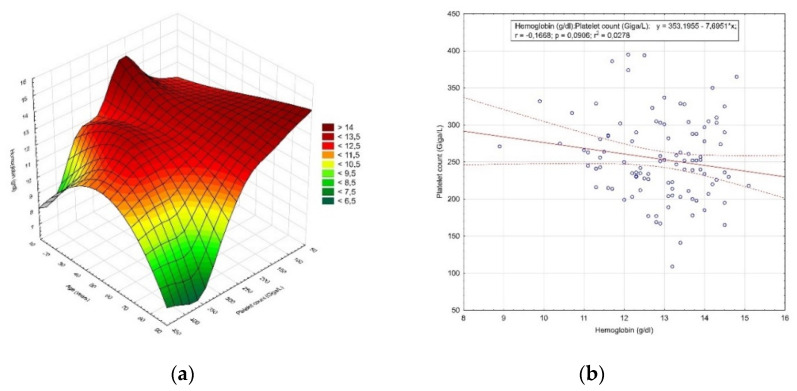
The results of the analysis of patients diagnosed with endometrial polyps. (**a**) Distribution of the hemoglobin concentration value and the number of platelets by age; (**b**) correlation between the hemoglobin concentration value and the number of platelets.

One hundred and four patients were diagnosed with endometrial polyps. The age range in this group was between 21 and 78 years (47.4 ± 12.9 years) (*p* = 0.079). In addition, 65.4% (68 women) were premenopausal, while 34.6% (36 women) were postmenopausal. The mean hemoglobin concentration in patients with endometrial polyps was 12.96 ± 1.18 g/dL (*p* = 0.017). Moreover, 20.19% (21 women) were below normal, of whom 19.23% (20 patients) were moderately anemic and 0.96% (1 patient) was moderately severe. For this group, the mean platelet count was 253.5 ± 54.49 Giga/L (*p* = 0.24) and 1.92% (2 patients) were below the normal range. The distribution of the hemoglobin concentration value and the number of platelets by age is shown in Figure 4a, while the correlation between the hemoglobin concentration value and the number of platelets is shown in Figure 4b.

### 3.4. Heavy Menstrual Bleeding

**Figure 5:**
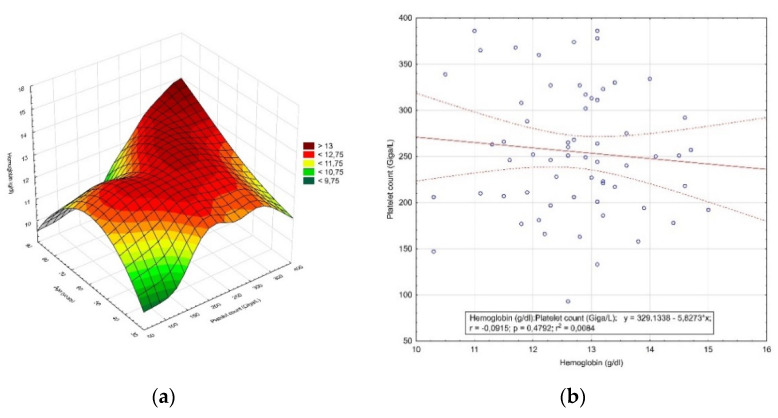
The results of the analysis of patients with heavy menstrual bleeding. (**a**) Distribution of the hemoglobin concentration value and the number of platelets by age; (**b**) correlation between the hemoglobin concentration value and the number of platelets.

The group of patients with heavy menstrual bleeding included 62 women, aged 29–56 (44.8 ± 6.11 years) (*p* = 0.288). In addition, 80.6% (50 patients) were postmenopausal and 19.4% (12 patients) were premenopausal. The average hemoglobin concentration in this group was 12.71 ± 1.07 g/dL (*p* = 0.481), 25.8% (16 patients) presented with mild anemia, and none of them had moderately severe anemia. The mean platelet count in this group was 255.08 ± 68.37 Giga/L (*p* = 0.385) and the results of 4.84% (3 patients) were below the reference values. The distribution of the hemoglobin concentration value and the number of platelets by age is shown in Figure 5a, while the correlation between the hemoglobin concentration value and the number of platelets is shown in Figure 5b.

### 3.5. Myomatosis

**Figure 6:**
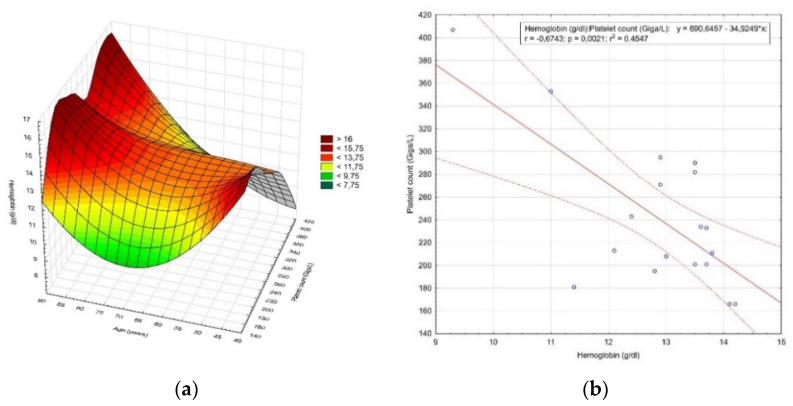
The results of the analysis of patients hospitalized due to myomatosis. (**a**) Distribution of the hemoglobin concentration value and the number of platelets by age; (**b**) correlation between the hemoglobin concentration value and the number of platelets.

Sixteen women were hospitalized due to myomatosis. Their age was in the range of 37–66 years (49.6 ± 8.4 years) (*p* = 0.025). In addition, 55.5% (10 women) were postmenopausal and 44.5% (8 women) were premenopausal. The mean value of hemoglobin concentration in this group was 12.86 ± 1.25 g/dL (*p* = 0.007). Two patients (12.5%) had moderate anemia, and none of them suffered from moderately severe anemia. The mean number of platelets was 241.67 ± 64.64 Giga/L (*p* = 0.053). None of the patients in this group had a platelet level below normal. The distribution of the hemoglobin concentration value and the number of platelets by age is shown in Figure 6a, while the correlation between the hemoglobin concentration value and the number of platelets is shown in Figure 6b.

## 4. Discussion

The results presented in this article show abnormalities in the level of hemoglobin and the number of platelets in women who have undergone hysteroscopy due to pathological uterine bleeding, endometrial polyps, endometrial hyperplasia, uterine myomatosis, and excessive menstrual bleeding. The total prevalence of these disorders was 24.63% (134 women). The most common disorder was a reduction in hemoglobin level. It occurred in 17.46% (95 patients), of which the majority (16.54%) was moderate anemia, which appeared in 90 patients. Moderately severe anemia was reported in 0.92% (5 patients). None of the patients experienced severe anemia. The decrease in hemoglobin was most common in the group of patients who underwent hysteroscopy, according to heavy menstrual bleeding.

Thrombocytopenia was less frequent, which in the whole group was 4.04%, and was most common among patients with endometrial hyperplasia. Elevated values of hemoglobin concentration appeared in 0.37% and had non-statistically significant values and a number of platelets above the reference range occurred in 1.47% of patients.

Relating the results to the PALM COEIN classification system, anemia most often occurred in the AUB-O category, while thrombocytopenia in the AUB-A category.

Anemia that is not treated for a long time can lead to serious complications, in extreme cases, even to multi-organ failure and death. Complications are more common in older adults and most often affect the cardiovascular system. In the younger population, anemia can lead to cognitive and developmental delays, and these complications are difficult to treat [20].

Thrombocytopenia occurs in approximately 5–10% of all postoperative patients. While the hemostatic function depends on many factors, platelet count and function are key components. The relationship between platelet count and bleeding risk is non-linear and depends on platelet function and other patient-specific factors. However, there are data from a large observational study in asymptomatic, non-hospitalized surgical patients without indications for coagulation studies, suggesting that there may be a relationship between preoperative thrombocytopenia and 30-day mortality [21]. According to these data and the results of the analysis, monitoring of the level of hemoglobin and platelets in patients qualified for the hysteroscopy procedure seems reasonable and necessary.

## 5. Conclusions

1. The majority of patients who underwent hysteroscopy due to pathological bleeding, endometrial polyps, endometrial hyperplasia, uterine fibroids, and excessive menstrual bleeding had normal hemoglobin concentration and platelet counts.
2. The most common disorder was moderate anemia, which occurred with the highest frequency in the group of patients who underwent hysteroscopy due to heavy menstrual bleeding.
3. None of the patients in the analyzed group developed severe anemia.
4. Thrombocytopenia was a rare disorder, most frequently seen in patients who underwent hysteroscopy, according to endometrial hyperplasia.
5. The least frequent phenomenon was elevated hemoglobin concentration.
6. In view of possible complications, it seems reasonable to determine hematological parameters, such as hemoglobin and platelet counts in patients referred for hysteroscopy for pathological uterine bleeding, endometrial polyps, endometrial hyperplasia, myomas, and heavy menstrual bleeding.

## Data Availability

Data available on request due to restrictions, because all patients were diagnosed and treated according to national guidelines and agreements.
